# Tailored 3D Agarose-Well Integrated with Human Skin Equivalents for Enhanced Skin Penetration Assessment

**DOI:** 10.3390/gels10110691

**Published:** 2024-10-24

**Authors:** Chaewon Woo, Jina Byun, Sung Gyu Shin, Heeseon Yoo, Sungwoo Cho, Donghun Lee, Taezoon Park, Jae Hyun Jeong

**Affiliations:** 1 Department of Chemical Engineering, Soongsil University, Seoul 06978, Republic of Korea; chaewon311@soongsil.ac.kr (C.W.); byunjina@naver.com (J.B.); whitegd45@soongsil.ac.kr (S.G.S.); hs2@soongsil.ac.kr (H.Y.); chosw1120@gmail.com (S.C.); 2 Department of Mechanical Engineering, Soongsil University, Seoul 06978, Republic of Korea; delee04@ssu.ac.kr; 3 Department of Industrial & Information Systems Engineering, Soongsil University, Seoul 06978, Republic of Korea; tzpark@ssu.ac.kr

**Keywords:** 3D agarose-well, skin penetration, human skin equivalents, high-performance gels

## Abstract

We developed a tailored 3D Agarose-well system integrated with reconstructed human skin equivalents to enhance skin penetration assessments. This system addresses common limitations in traditional trans-well reconstructions, such as dermal layer contraction and limited lateral diffusion, by entangling collagen fibrils within the Agarose-well. We evaluated the penetration behavior of three peptides, with and without skin-penetrating peptide (SPP) sequences, alongside adenosine, a known anti-wrinkle agent. Despite a SPP having a molecular weight approximately four times greater than that of adenosine, its kinetic constant was similar, with values of about 39 and 34, respectively. Moreover, this living skin equivalent system not only allowed for the evaluation of adenosine penetration, but also demonstrated its biological effects, with adenosine significantly enhancing procollagen synthesis by approximately 23% compared to the control. Overall, this novel strategy holds the potential for tailoring 3D Agarose-wells and advancing high-performance gel development, making it a promising approach for applications in tissue engineering, medical science, regenerative medicine, and cosmetics.

## 1. Introduction

The skin, as the body’s largest organ, acts as a primary barrier against environmental aggressors while regulating substance absorption. Its complex structure comprises the epidermis, dermis, and subcutaneous layers, each contributing to the skin’s overall barrier function. Notably, understanding how various compounds penetrate this barrier is essential for applications in dermatology, toxicology, and cosmetic science [1,2,3,4]. In recent years, the development of in vitro skin models has become indispensable for studying skin penetration and assessing the biological effects of topically applied compounds. Traditional methods, such as excised animal or human skin, have been used extensively; however, their application is limited by the inherent variability and non-living nature of these tissues, which impairs the evaluation of long-term biological effects. Consequently, reconstructed human skin equivalents, which closely mimic in vivo skin physiology, have emerged as valuable tools, offering more reliable and reproducible models for penetration studies [5,6].

However, conventional systems for culturing reconstructed human skin equivalents, such as trans-well plates and Franz diffusion cells, present significant limitations. Trans-well systems often lead to contraction of the dermal layer and restrict lateral diffusion, resulting in poor structural integrity and inaccurate assessment of penetration behavior. Similarly, the Franz diffusion cell, while widely used, typically employs non-living excised skin, making it unsuitable for evaluating dynamic biological responses post-penetration [7,8,9]. These limitations have driven researchers to explore alternative methods for improving the accuracy and relevance of skin penetration studies.

Hydrogels, particularly those based on agarose, have gained attention for their tunability, structural support, and compatibility with biological systems. Agarose, a naturally derived polysaccharide, is known for its biocompatibility, low toxicity, and ability to form stable gel matrices that support tissue-like structures. Recent innovations have focused on modifying agarose with alkyl groups to tailor its physical properties, thereby creating hydrogels that better mimic the mechanical properties of the skin [10]. Such modifications have led to the development of advanced systems like the 3D Agarose-well, which addresses many of the limitations posed by traditional culture methods [11].

**Figure 1:**
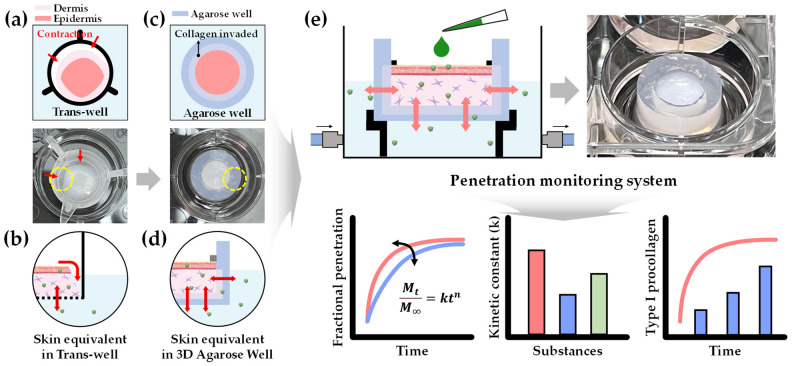
Scheme of the penetration monitoring system utilizing a living skin equivalent reconstructed in the 3D Agarose-well. (**a**) The skin equivalent in the trans-well detached from the insert due to cellular contraction. (**b**) Substances directly enter the contracted area without penetrating the skin model. (**c**) The skin equivalent integrated with the 3D Agarose-well retains its shape due to the formation of an interpenetrating network, facilitating the infiltration of substances into the skin equivalent without additional pathways. (**d**) The monitoring system continuously tracks the penetration behavior of substances under sink conditions. (**e**) Final monitoring system scheme utilized for penetration, kinetic constant, and procollagen production analysis.

In this study, we introduce a tailored 3D Agarose-well system designed to integrate seamlessly with reconstructed human skin equivalents. The term, tailored 3D Agarose-well, refers to the ability to adjust the physical properties of the dermal layer by modifying the ratio of alkyl-grafted agarose, allowing for more precise control over structural and diffusion characteristics. This system improves upon previous methods by preventing dermal layer contraction, providing a more stable environment for skin penetration studies, and facilitating the accurate measurement of substance diffusion across living skin equivalents. By modifying the hydrogel properties, this system supports the entanglement of collagen fibrils within the gel matrix, preserving the architecture of the skin equivalent and enabling continuous monitoring of substance penetration (Figure 1).

To validate the system, we investigated the penetration behavior of several compounds, including peptides with and without skin-penetrating peptide (SPP) sequences, and adenosine, a known anti-wrinkle agent. These experiments demonstrated that the tailored 3D Agarose-well system provides consistent and reliable penetration data, while also allowing for the evaluation of biological effects such as procollagen synthesis, an important marker for anti-aging efficacy.

The tailored 3D Agarose-well system represents a promising advancement in skin penetration research, providing a platform for more accurate, reliable, and biologically relevant assessments [12,13]. Beyond skin penetration studies, this system holds potential for broader applications in tissue engineering, medical science, regenerative medicine, and cosmetics, particularly in the development of high-performance gels tailored to specific research needs.

## 2. Results and Discussion

### 2.1. Human Skin Equivalents Reconstructed in 3D Agarose-Well

**Figure 2:**
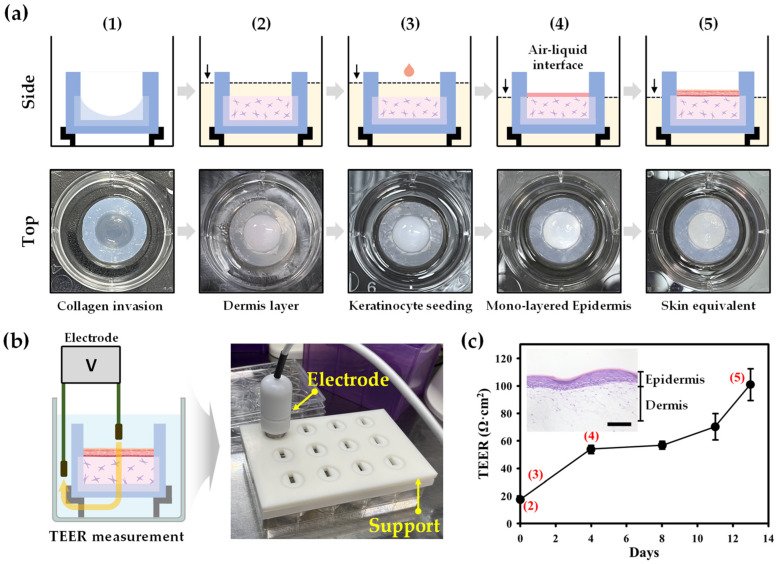
(**a**) The entire process of reconstructing the skin equivalent in the 3D Agarose-well. Initially, the collagen solution infiltrated the 3D Agarose-well (**a-1**), followed by the addition of the dermal cell suspension to prepare the dermal layer (**a-2**). Keratinocytes were cultured on the dermal layer (**a-3**) and exposed to air to form a monolayer for differentiation (**a-4**). The full-thickness skin equivalent was then reconstructed within the 3D Agarose-well (**a-5**). (**b**) The reconstruction processes were monitored using TEER measurements at each step, including the formation of the keratinocyte monolayer. (**c**) The TEER values at each step are presented in the graph, starting from the completion of the dermal layer designated as day 0. (Scale bar: 100 μm).

Figure 2 illustrates the preparation process of the skin equivalent within the Agarose-well. Initially, a collagen solution was introduced into the Agarose-well, allowing for uniform diffusion throughout the matrix (Figure 2a). Following this, a dermal layer containing human dermal fibroblasts was established, creating an interpenetrating polymer network. Keratinocytes were then seeded onto the dermal layer, and the corneous layer was differentiated from the epidermis through an air–liquid interface culture method, culminating in the successful reconstruction of the skin equivalent. The optimal timing for initiating air–liquid interface culture was determined by measuring transepithelial electrical resistance (TEER). Traditional methods for constructing skin equivalents typically utilize trans-well systems. While effective for forming dermal and epidermal layers, these systems often lead to contraction of the skin equivalent due to cell proliferation. In contrast, the 3D Agarose-well, as depicted in Figure 1, maintained its structural integrity even during complete cell proliferation. This stability can be attributed to the entanglement of collagen fibrils within the Agarose-well, resulting in a robust skin equivalent.

The skin equivalent was developed to include a dermis, epidermis, and a differentiated corneum. To achieve corneum differentiation, keratinocytes must be exposed to air once they fully cover the dermis layer. In this study, we ensured the density of the keratinocytes by examining the transepithelial electrical resistance (TEER) values at each stage of development. The resistance of the skin equivalent was calculated by subtracting the resistance of an empty trans-well from the total resistance measured in the trans-well containing the skin equivalent:

(1) $$TEER=R_{Tissue}\left(\Omega \right)\times Area\left({cm}^{2}\right)$$

where *R_Tissue_* is the resistance of the skin equivalent, and area is the dimension of the skin equivalent. For the TEER measurements, we used five skin equivalents for each value, with a trans-well area of 4.25 cm^2^.

Figure 2c displays the TEER values at each step, from the dermis layer to the fully covered dermis layer with differentiated keratinocytes forming the corneum layer. The TEER value for the dermis layer was recorded at 18 Ω·cm^2^. Following the seeding of keratinocytes, the TEER value increased to about 55 Ω·cm^2^ as the keratinocytes fully covered the dermis. At this stage, the epidermis began air–liquid interface culture to promote keratinocyte differentiation. The TEER value gradually increased as differentiation progressed, reaching around 100 Ω·cm^2^ after 13 days. The H&E-stained images embedded within the TEER graph (Figure 2c) depict the skin equivalent’s structural changes over time. Four days after initiating the air–liquid interface culture, the dermis layer measured approximately 0.35 cm in thickness, while the epidermis layer was about 30–40 μm thick. After 13 days, the epidermis thickness increased to approximately 100 μm. Although the epidermis layer was roughly 35 times thinner than the dermis layer, its TEER value increased about five-fold compared to the dermis due to the differentiation of keratinocytes into a multilayered structure. Furthermore, the TEER value significantly increased after 13 days of cultivation, reflecting the development of tight junctions within the corneum layer. Compared to other skin models, such as those utilizing fibrin or collagen alone, the 3D Agarose-well allows for more controlled differentiation of keratinocytes, as evidenced by the TEER values and histological analyses. The stable TEER values and robust structural differentiation in our system underscore its advantage in maintaining long-term culture viability.

### 2.2. Enhanced Skin Penetration Assessment Using the Tailored 3D Agarose-Well

**Figure 3:**
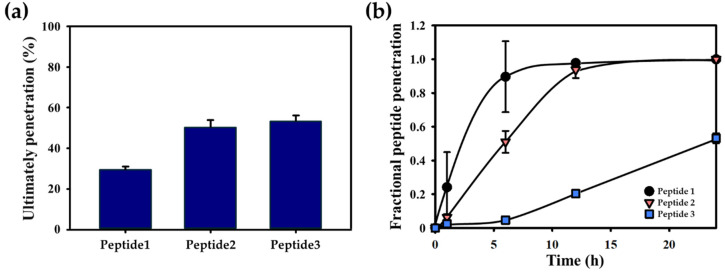
(**a**) The ultimate penetration (${(M}_{\infty }/M_{initial})\times 100$) of peptides was determined by comparing the initial concentration of peptides in the suspension to the concentration remaining after penetration. (**b**) The fractional peptide penetration over time ($M_{t}/M_{\infty }$) was calculated by dividing the concentration of peptides remaining after each time point by the final penetration value. Mean values and standard deviations were calculated from five replicates (*n* = 5).

The tailored 3D Agarose-well system was utilized to evaluate peptide penetration and the factors influencing it. In this study, adenosine and three peptides were applied to the surface of the skin equivalent, and their penetration behavior was examined by measuring peptide concentrations in the receptor medium beneath the skin equivalent and agarose layer (Table 1). Figure 3a illustrates the final penetration levels ${(M}_{\infty }/M_{initial})\times 100)$ for each peptide, while Figure 3b presents their fractional penetration ($M_{t}/M_{\infty })$ over time. Peptides 1 and 2, which contain skin-penetrating peptide (SPP) sequences, exhibited a faster and more efficient penetration than peptide 3, which lacks these sequences and has roughly half the molecular weight of peptides 1 and 2. Peptides 1 and 2 fully penetrated the skin within 24 h, while peptide 3 continued to release beyond that time frame, highlighting the enhanced penetration capabilities of peptides 1 and 2. This observation echoes the results from the previous study [14], which examined the SPACE peptide—similar in sequence to our peptide 1—and its ability to facilitate the delivery of macromolecules, such as Cyclosporine A (CsA), through excised human skin using a Franz diffusion cell. Notably, the mechanism of action involved the interaction between the SPPs and skin proteins, leading to changes in secondary protein structures that favorably influenced the skin’s permeability. The transcellular pathway enhanced by the SPPs promoted efficient partitioning into keratin-rich corneocytes, facilitating drug delivery. Our results further substantiate these findings, demonstrating that peptides 1 and 2 achieved full penetration of the skin within 24 h, while peptide 3 continued to release beyond this timeframe, underscoring the enhanced penetration capabilities of the SPP-containing peptides. The implications of these findings are significant, as they suggest that the mechanism of SPPs, particularly those resembling the SPACE peptide, plays a critical role in mediating effective drug delivery through the skin.

**Figure 4:**
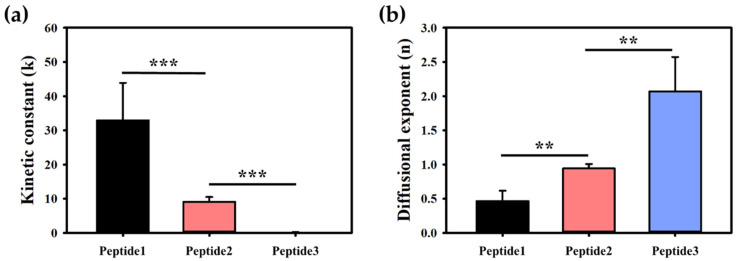
Penetration factors of model peptides. (**a**) Kinetic rate constant and (**b**) diffusion exponent. A higher kinetic rate constant was observed when the initial fraction of penetration increased rapidly, whereas a lower diffusion exponent indicated dominant diffusion behavior. Statistical significance levels were established as follows: ** *p* ≤ 0.01, and *** *p* ≤ 0.001. Mean values and standard deviations were calculated from five replicates (*n* = 5).

**Figure 5:**
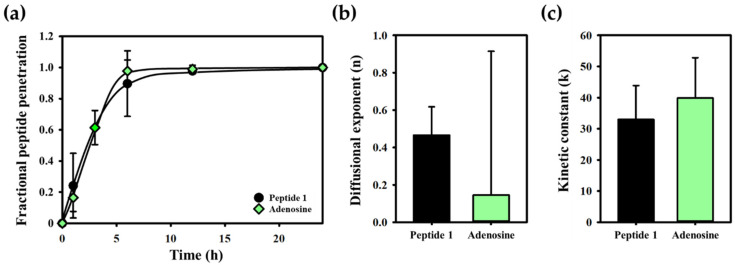
The penetration factors compared peptide 1 and adenosine; (**a**) fraction of peptide penetration; (**b**) diffusion exponent; (**c**) kinetic constant. The penetration factors of peptide 1; fraction of penetration, diffusion exponent, and kinetic constant were approximated comparing adenosine even though peptide 1 has 4 times more molecular weight. Mean values and standard deviations were calculated from five replicates (*n* = 5).

To further quantify the penetration dynamics, the kinetic rate constant (k) and diffusional exponent (n) were calculated using Equation (2) illustrated in the Materials and Methods section. Model peptide 1 had the highest kinetic rate constant at 33.4, followed by peptide 2 at 9.6, and peptide 3 at 0.11 (Figure 4a). The diffusional exponent for model peptide 1 was the lowest at 0.49, followed by peptide 2 at 0.96, and peptide 3 at 2.07 (Figure 4b). A lower diffusional exponent signifies diffusion-dominated transport, while a higher exponent suggests a relaxation-controlled mechanism. Comparing these values, model peptide 1, which had the most effective penetration profile, was compared with adenosine, a smaller molecule with a molecular weight approximately four times lower (Figure 5). Despite model peptide 1’s larger size, its penetration characteristics, including fractional penetration and kinetic rate constant, were comparable to adenosine. This demonstrates the capability of the 3D Agarose-well system to accurately assess and compare the penetration properties of peptides.

**Figure 6:**
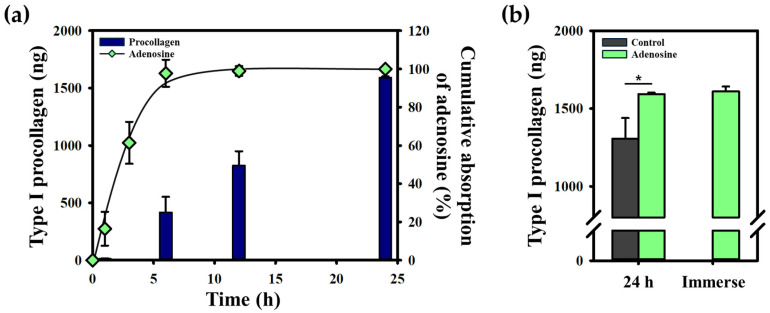
The bright images show that adenosine activates procollagen synthesis. (**a**) Synthesized type I procollagen and remaining adenosine were detected on the medium at each time. (**b**) The synthesis of type I procollagen was higher in the treated adenosine group compared to the untreated group. This penetration system demonstrates the successful penetration of adenosine compared to the amount of type I procollagen in the immersed group, which was approximated. Statistical significance levels were established as follows: * *p* ≤ 0.05. Mean values and standard deviations were calculated from five replicates (*n* = 5).

The living diffusion system, composed of a full-thickness skin equivalent with both dermal and epidermal layers, offers a robust platform for the simultaneous evaluation of skin penetration and biological effects, particularly those associated with dermal remodeling and anti-aging mechanisms. In this study, adenosine—a compound widely recognized for its role in anti-aging formulations due to its capacity to stimulate collagen production—was employed to assess its bioactivity, focusing specifically on type I procollagen synthesis. Previous studies have highlighted the significance of adenosine in enhancing skin elasticity and hydration and reducing visible signs of aging, such as fine lines and wrinkles [15,16]. These properties make it an ideal candidate for examining the effectiveness of the 3D Agarose-well system as both a penetration assessment tool and a bioactivity monitoring platform. To quantify procollagen Type I synthesis, we used the Procollagen Type I C-peptide (PIP) EIA kit, a reliable method for measuring collagen precursors. Adenosine’s bioactivity was evident, as it triggered fibroblast activity in the dermal layer, resulting in enhanced procollagen production. This was measured in the receptor medium, indicating that adenosine successfully penetrated the skin barrier and stimulated fibroblast-mediated procollagen synthesis. Figure 6a illustrates the penetration timeline of adenosine and its subsequent biological effect on procollagen production. Notably, even after complete adenosine penetration within the first 15 h, procollagen synthesis continued, suggesting that the compound remains active in the skin for an extended period. Procollagen synthesis increased by approximately 23% in comparison to the control group, which was cultured in a standard medium without adenosine supplementation (Figure 6b). These results underscore the dual function of the living diffusion system: not only can it measure the efficiency of skin penetration, but it also allows for real-time monitoring of bio-effects, such as extracellular matrix remodeling through collagen production.

In addition to penetration efficacy, this system facilitates a deeper understanding of how the penetrated molecules interact with the skin at a cellular level. Adenosine’s ability to promote procollagen synthesis beyond the point of penetration suggests that it may remain in active form, continuously engaging with dermal fibroblasts to stimulate collagen production over time. This prolonged activity is particularly beneficial for evaluating compounds intended for long-lasting cosmetic or therapeutic effects. The sustained increase in TEER values during this period also corroborates the role of adenosine in enhancing skin barrier function, likely through improved cellular cohesion and the formation of tight junctions in the epidermal layers.

Moreover, Figure 6b presents a direct comparison of procollagen synthesis between the penetration system and a traditional immersion method, where skin equivalents were immersed in an adenosine solution at the same concentration for 24 h. Interestingly, the results revealed no significant difference in procollagen production between the two methods. This finding suggests that the 3D Agarose-well system is not only comparable to traditional trans well and immersion-based systems, but also provides a more physiologically relevant environment for testing. The skin equivalent in the Agarose-well maintains structural integrity and viability over time, thereby preserving the functionality of living cells and tissues. This underscores the value of using the Agarose-well system for studies requiring long-term assessment of both penetration and cellular responses.

In conclusion, the 3D Agarose-well system offers significant advantages over conventional skin models. It allows for a more realistic simulation of in vivo conditions, enabling accurate penetration studies coupled with an evaluation of downstream biological effects. The ability to monitor both skin barrier function and cellular activity in a living system provides a comprehensive approach to studying compounds such as adenosine, whose effects extend beyond mere penetration. Future studies could further explore the use of this system with other bioactive compounds, potentially opening new avenues for research into therapeutic agents, cosmetic formulations, and drug delivery systems targeting the skin. By bridging the gap between skin penetration and functional biological outcomes, the Agarose-well system represents a significant advancement in the field of dermal research.

## 3. Conclusions

In this study, we developed a tailored 3D Agarose-well system to improve the structural stability of full-thickness human skin equivalents and enhance the accuracy of skin penetration assessments. The system prevented skin equivalent contraction and enabled effective diffusion measurements. Peptides with skin-penetrating sequences showed significantly higher penetration rates compared to smaller peptides lacking these sequences. Additionally, adenosine, a common anti-aging ingredient, not only penetrated effectively but also stimulated procollagen synthesis in dermal fibroblasts, demonstrating the system’s ability to evaluate both penetration behavior and bio-effects. The Agarose-well system thus provides a robust, living model for studying the interaction between topical substances and human skin, offering a valuable tool for both cosmetic and pharmaceutical research.

## 4. Materials and Methods

### 4.1. Materials

Collagen solution (Type I, 6 mg/mL) was obtained from Advanced BioMatrix (San Diego, CA, USA). Agarose, sodium hydroxide, sodium bicarbonate, 4-(2-hydroxyethyl)-1-piperazineethanesulfonic acid (HEPES), and adenosine were sourced from Sigma-Aldrich (St. Louis, MO, USA). Bioactive peptides were provided by PurioGen, Inc. (Seoul, Republic of Korea). Peptides 1 and 2 contain skin-penetrating peptide (SPP) sequences, while peptide 3 does not. Human Dermal Fibroblast (neonatal, HDFn) and Human Epidermal Keratinocyte (neonatal, HEKn) cells were purchased from ATCC (Manassas, VA, USA). M106 medium, Epilife^®^, Human Keratinocyte Growth Supplement (HKGS), and Low Serum Growth Supplement (LSGS) were obtained from Gibco (Grand Island, NY, USA).

### 4.2. Human Skin Equivalents Reconstructed in 3D Agarose-Wells

Agarose-wells were prepared using a 1.0% (*w*/*v*) agarose aqueous solution. Agarose was dissolved in deionized (DI) water at 121 °C for 20 min. The solution was poured into molds produced by a stereolithography apparatus (SLA), sterilized by immersing in 70% ethanol for 15 min and subsequently exposed to UV light for 30 min. The agarose mixture was allowed to cool at room temperature, polymerizing to form the wells. Once set, the Agarose-wells were removed from the molds and stored in PBS until use [11]. Then, human skin equivalents were reconstructed in the 3D Agarose-wells. First, the wells were dried until approximately 80% of the moisture content was removed. A prechilled 500 μL collagen solution was added to the dried wells and incubated at 4 °C overnight for absorption. The dermis layer was reconstructed by mixing 400 μL of collagen solution with 100 μL of buffer (composed of sodium bicarbonate, HEPES, and sodium hydroxide) and 10,000 HDFn cells/mL. This mixture was added to the wells and incubated for 1 h at 37 °C and 5% CO_2_. The dermis was cultured in M106 medium supplemented with LSGS, replaced every 2 days. The epidermis layer was developed by seeding HEKn cells (1.0 × 10^5^ cells/cm^2^) onto the dermis and incubating for 1 h. After forming a monolayer, the culture was transitioned to an air–liquid interface for differentiation. Fresh medium was replaced outside the wells every 2 days.

### 4.3. Structural Integrity of the Skin Equivalent Evaluated by TEER

The integrity of the epidermal layer was evaluated using transepithelial electrical resistance (TEER), measured with an Epithelial Volt/Ohm Meter (EVOM3, World Precision Instruments, Sarasota, FL, USA) [17,18]. Before TEER measurements, electrodes were sterilized with 70% ethanol and UV light. PBS was added to the Agarose-wells, and the electrodes were positioned over the skin equivalents. TEER values were recorded over 13 days during air–liquid interface culture. H&E staining was used to observe skin morphology in relation to TEER results.

### 4.4. Skin Penetration Monitoring Using a Living Human Skin Equivalents

The 3D Agarose-wells were integrated into a diffusion system for in vitro skin penetration studies, allowing for both side and bottom diffusion. Polydimethylsiloxane (PDMS) O-rings were employed to guide penetration from the surface and prevent diffusion into the Agarose-wells. Peptide suspensions (100 mg/mL) were applied to the skin equivalents, and receptor medium was collected at 0, 3, 6, 12, and 24 h intervals. Samples were analyzed using High-performance Liquid Chromatography (HPLC). The penetration kinetics were determined using the Ritger–Peppas equation:

(2) $$\frac{M_{t}}{M_{\infty }}=kt^{n}$$

where *M_t_*/*M_∞_* is a fraction of peptide penetration; *M_t_* is the number of peptides penetrated through the skin equivalent at the time; *t* is the time when collecting medium; *M_∞_* is the number of peptides penetrated through the skin equivalent until the end; *k* is the kinetic rate constant; and *n* is the diffusional exponent related to the penetration mechanism [19,20,21]. The ultimate penetration was defined from the following equation:

(3) $$Ulitimately\;penetration=\frac{M_{\infty }}{M_{i}}\times 100\left(\%\right)$$

where *M_i_* is the number of peptides released on the skin equivalent when the penetration test begins.

### 4.5. Evaluation of Procollagen Synthesized in Skin Equivalent

In vitro enzyme immunoassay was performed using the Procollagen Type I C-peptide (PIP) EIA Kit (MK101, TAKARA, Kusatsu, Japan). The culture medium from the skin equivalents was mixed with a monoclonal antibody solution and incubated in an anti-PIP monoclonal antibody-coated plate at 37 °C for 3 h. After washing with PBS, a substrate solution (TMBZ solution, 3,3′,5,5′-tetramethylbenzidine) was added to develop color at room temperature for 15 min. Subsequently, a stop solution was added to stabilize the reaction, and the optical density was measured using a microplate reader (Thermo Scientific, Waltham, MA, USA) at 450 nm. A standard curve for the quantification of type I procollagen was generated according to the protocol. Absorbance measurements of standard solutions ranging from 0 to 640 ng/mL were used to construct the standard curve. Based on this curve, the concentration of type I procollagen in the samples was quantified.

### 4.6. Statistical Analysis

Experimental data are presented as mean ± standard deviation (S.D.). Statistical comparisons were made using Tukey’s Test. Significance levels were set at * *p* ≤ 0.05, ** *p* ≤ 0.01, and *** *p* ≤ 0.001.

## Figures and Tables

**Table 1:** Characterization of the SPPs and adenosine.

Sample	Sequence	M.W. (g/mol)
Model peptide 1	ACTGS-TQHQC-G	1092
Model peptide 2	HIITD-PNMAE-YL	1417
Model peptide 3	Pal-GHK	579
Adenosine	-	267

## Data Availability

The original contributions presented in this study are included in the article, further inquiries can be directed to the corresponding author.
